# Expression of Concern: LncRNA *TUG1* promotes the progression of colorectal cancer via the miR-138-5p/ZEB2 Axis

**DOI:** 10.1042/BSR-20201025_EOC

**Published:** 2021-04-06

**Keywords:** biological function, CRC, miR-138-5p, TUG1, ZEB2

The Editorial Office has been made aware of potential issues surrounding the scientific validity of this paper, hence has issued an expression of concern to notify readers whilst the Editorial Office investigates.

